# Viroids: “living fossils” of primordial RNAs?

**DOI:** 10.1186/s13062-016-0116-7

**Published:** 2016-03-25

**Authors:** Theodor O. Diener

**Affiliations:** Department of Cell Biology and Molecular Genetics, University of Maryland, 6141 Plant Sciences Building, College Park, MD 20742 USA

**Keywords:** Introns, RNA World, Abiogenesis, LUCA (the Last Universal Common Ancestor)

## Abstract

The discovery of the viroid in 1971, which initiated the third major expansion of the biosphere towards smaller living entities—after discovery of the “subvisual” microorganisms in 1675 and that of the “submicroscopic” viruses in 1892—has been officially endorsed by the International Committee on Virus Taxonomy as a new order called *subviral agents*.

In 1989, I proposed that, based on their respective molecular properties, viroids are more plausible “living fossils” of the hypothetical RNA World (widely assumed to have existed prior to the evolution of DNA or proteins) than are intron-derived RNAs, which were, at that time, suggested as putative survivors. There were few citations of my proposal—and virtually none of viroids—beyond plant virology unil 1994, when Cheles-Flores critically examined the hypothesis and pointed out a serious difficulty, as well as a process by which this difficulty could be overcome. In 2013, when investigations by Koonin and Dolja revealed that of extant RNAs, viroids “strikingly” display some of the molecular properties posited for the earliest evolving, selfish RNAs (primordial RNAs), but, because extant organisms, aside from higher plants, appear not to harbor viroids, they cannot be regarded as primordial fossils, but appear to have evolved post LUCA (the Last Universal Common Ancestor). Here, I review whether some evidence nevertheless is compatible with the original postulate of the 1989 hypothesis. My analysis reveals no unequivocal evidence for an ancient origin of viroids, but suggests, alternatively, that viroids may have evolved de novo more recently, probably by novel processes similar to those suggested by each reviewer.

These results are important, because they help illuminate a little understood period of abiogenesis—after the abiotic synthesis of life’s chemical building blocks, which is, in principle, understood, and before the evolution of DNA and proteins in the late RNA World.

## Background

The discovery of the viroid in 1971 [1] initiated the third major extension of the biosphere to include smaller living entities—after the discovery of the “subvisible” microorganisms by Antonie van Leeuwenhoek in 1675 and the “submicroscopic” viruses by Dmitri Iosifovich Ivanovsky in 1892. It has been recognized by the International Committee for Virus Taxonomy with the creation of a new order of *subviral agents* [2, 3]. Beyond plant virology, recognition of the new order was relatively slow and, even today, subviral agents are often wrongly conflated with large, noncoding RNAs (lncRNAs) [4–7] because of their similar sizes (equivalent to considering humans as “life-sized mannequins with life”), in disregard of the fundamental fact that lncRNAs are lifeless transcripts from cellular DNA, whereas viroids, like viruses, are exogenous, autonomously replicating RNAs—which, with lengths of 246 to 401 nucleotides, are at the frontier of life [8].

## The hypothesis

In 1989, I hypothesized [9] that, based on their respective molecular properties, viroids are more plausible “living fossils” of the RNA World, than are intron-derived RNAs, which were then so considered [10, 11].

## Testing of the hypothesis

Here, I report how the biological community reacted to my hypothesis and by what means scientists suggested to test its plausibility.

While my hypothesis was frequently cited in the biological literature with little or no comment, it was first critically examined in 1994 by Cheles-Flores [12], who pointed out that “a difficulty may be raised against the Diener hypothesis that viroids may be interpreted as molecular fossils of the RNA world,” in that viroids are known to exist only in angiosperms, whose first appearance was in the Cretaceous period. The author presented a scheme, based on cyanobacteria, which, after “extensive additional work by plant pathologists”, if successful, would “remove the importance, in the preservation of the relics of the RNA world, of the time of the first appearance of angiosperms” and thus show that “viroids could have been present during the major part of the duration of life on Earth.”

In a second 1994 paper [13], Chela-Flores expanded on these thoughts, but again asked the question whether it is possible to envisage a possible evolutionary pathway of the early replicators spanning the vast time span separating the first appearance of the angiosperms, late in the Mesozoic era (the Lower Cretaceous) from the most likely sub-eras in which the RNA world may have occurred, namely the Hadean/Early Archean. The author suggested that “through horizontal gene transfer, as well as through a series of symbioses in the precursor cells of the land plants, the genes of the replicases associated with RNA plasmids and other putative DNA-independent RNA replicators may have been transferred vertically, eventually becoming specific to the angiosperms.” However, no further report from Cheles-Flores has appeared; apparently, the proposed experimental work has not been performed or it was not successful.

In 1998, Jeffares. et al. [14] reported results of a theoretical study, in which the authors estimated—based on what they considered to be plausible parameters—which of many individual, extant RNAs may be relics of the RNA World and which are probably of more recent provenance. While the authors cited my 1989 hypothesis (as described in a 1993 book chapter [15]), they do not discuss it, or its relevance to their work.

Given the strong trend RNA --- > RNP --- > protein, Jeffares et al. examined the phylogenetic distribution of RNA in modern organisms for relics and thus developed a model for complexity in the RNA world. Candidates were RNAs which fit at least one of their criteria: “catalytic, ubiquitous (or at least conserved within the eukaryotic lineage…), or central to some aspect of metabolism.”

Most importantly, in the context of the present assessment, is Jeffares et al.’s conclusion that viroids, plant satellite RNAs, and “hammer heads” are, indeed, ancient relics of the RNA world. It is not clear, however, on which of the parameters this conclusion is based. Viroids are listed confusingly (together with “hammer heads”) at the bottom of table 2—titled “RNA functions in modern cells”— under the rubric “Function,“ as “Various,” under the rubric “Distribution” as “Plant satellite RNA,“ and under the rubric “In the RNA world?” as “In RNA world (see text)” but this referral in a footnote is not illuminating.

By the authors’ parameters, many, if not most, extant RNAs (or their precursors) were also already present in the RNA world, including precursors of the three major cellular RNAs: rRNA, mRNA, and tRNA.

A distinction must be made, however, between Jeffares et al.’s chosen criteria for relics and those chosen in my 1989 publication. Whereas Jeffares et al. developed their “model for the *final* complexity of the RNA World,”— just prior to the evolution of translation and proteins—it was *actual* properties of viroids, listed in 1989, which I considered to suit them for survival in a prebiotic “soup” far less hospitable than that envisioned by Jeffares et al., i.e., for an earlier stage in the RNA World.

Therefore, if correct, Jeffares et al.‘s results would not only be in accord with the relic hypothesis, but more accurately define the stage of the RNA World, in which viroids (or their precursors) could presumably have prospered. However, Jeffares et al.’s choice of parameters is, by necessity, subjective and any substitutions would likely alter the conclusions. Even given the existence of an ancient RNA World, there are problems with understanding how, without DNA or proteins, cellular life could have evolved. One of the major problems is the question as to how one and the same kind of RNA molecule could serve simultaneously as both information carrier and biocatalyst, which would require a combination of features: good “templating” ability (for replication) and stable folding (for ribozymes). This poses a paradox, because well folded sequences are poor templates for copying, but poorly folded sequences are unlikely to be good ribozymes [16].

In 2013, Ivica et al. [16] described a novel strategy to overcome this dilemma; it is based on G:U wobble pairing in RNA: Unlike Watson-Crick base pairs, wobble pairs contribute highly to the energetic stability of the folded structure of their sequence, but only slightly, if at all, to the stability of the folded reverse complement. Sequences in the RNA World might therefore combine stable folding of the ribozyme with an unstructured, reverse-complementary genome, resulting in a ‘division of labor’ between the strands.

The investigators demonstrated this strategy by use of computational simulations of RNA sequences (including 40 viroid sequences) and their folding as experimental models of early replication, “involving non-enzymatic, template-directed, RNA primer extension.” The investigators recognized the fact that “interestingly, viroid RNA sequences…show significant asymmetry in folding energy between the infectious (+) and template (-) strands due to G: U pairing, suggesting that this strategy may even be used by replicators in the present day world,” as well, as postulated, in the RNA world. If so, this viroid-suggested process should be amenable—beyond computer simulation—by experimentation with actual RNA molecules.

Also in 2013, Ma et al. [17] cited my 1989 paper and—“inspired by features of viroids,”—studied their properties in mathematical simulations. Ma et al. were particularly interested in determining whether the known structure of viroids, “their circularity and small, self-splicing ribozymes (e.g., the hammerhead ribozymes), could have been instrumental in helping them overcome problems in replication and stability.” Their study indicated “that an RNA chromosome can spread (increase in quantity and be sustained) in the system, if it is a circular one and its linear ‘transcripts’ are readily broken at the sites between genes; the chromosome works as genetic material and ribozymes ‘coded’ by it serve as functional molecules.” Ma et al. concluded that circularity and self-cleavage are important for the spread of the chromosome.” The authors concluded that “in the RNA world, circularity and self-cleavage may have been adopted as a strategy to overcome the immediate difficulties for the emergence of a chromosome (with linked genes).” While Ma et al. thus seemed to provide important evidence for the possible ancient nature of viroids, their conclusions are placed in doubt by the unknown significance of mathematical simulations to real-world evolutionary situations.

Forterre’s revolutionary proposal [18] to divide the biosphere according to organisms’ fundamental properties into two parts: capsid-encoding organisms (i.e., viruses) and ribosome-encoding cellular organisms. The author’s proposal is compatible with my 1989 hypothesis, except that viroids and other subviral agents belong to neither part, but must be accorded a new, third part, consisting of non-capsid, non-cellular life forms.

Theoretical studies [19] indicate that “selfish replicons (genetic parasites) inevitably emerge in any sufficiently complex evolving ensemble of replicators.” Indeed, genetic parasites seem to be truly ubiquitous: some such elements apparently are associated with all cellular life forms and mathematical models of the evolution of replicator systems—aimed at the reconstruction of the first stages in the history of life—invariably reveal partitioning into hosts and parasites [19]. It is therefore not surprising, that viroids, if viewed as survivors of the RNA World, would not be self-replicating, but would, like viruses, depend on host enzymes for their (autonomous) replication.

Koonin and Dolja [20] studied “the evolutionary relationships between typical viruses with different replication-expression strategies and capsidless genetic elements,” on the basis of which they proposed a paradigm of virus-world evolution that is in accord with Forterre’s model. The authors stated that “host-parasite arms races are a major formative factor in all evolution of life” and that “the simplest genomic parasites might be small RNA molecules that encoded no proteins and consisted primarily of *cis* signals for replication.” Koonin and Dolja [20] also described features of hepatitis delta virus (HDV), which “appears to be a derivative of a viroid that encodes a protein required for replication and virion formation, and is encapsidated into particles that consist of the capsid protein of the helper Hepatitis B virus” and that “most likely HDV evolved from a viroid-like ancestor by acquiring a protein-encoding gene from a still unknown source and adapting to use the capsid protein of the helper virus.”

Koonin and Dolja [21] concluded from a landmark, comprehensive review of all virus groups, that “among the parasites of modern organisms, viroids that cause many diseases of plants and satellites of plant RNA viruses show a striking resemblance to the putative primordial parasites.” However, “given that viroids so far have been identified only in plants,” the authors considered it “unlikely that viroids are direct descendants of the primordial parasites.” But then, the authors stated again: “Nevertheless, viroids seem to recapitulate the principle features of the selfish elements from the ancient RNA world”—thus leading to an internal contradiction, in that by one criterion (molecular properties), viroids “strikingly” appear to be descendants of primordial RNAs, whereas by another criterion (apparent evolutionary age), they clearly are not. Which is correct?

## Implications of the hypothesis

Properties of Hepatitis delta virus may supply a clue: While the origin of HDV is unknown and while several different theories of its origin have been proposed [22], HDV RNA is almost universally considered (including by Koonin and Dolja [21]) to be the product of joining a viroidlike RNA with a protein-coding RNA. A possible extant biochemical process for such a joining has been proposed in 1996 [23]. Because no similarly joined RNAs have been found in hosts other than animals, it appears that the joining probably occurred post LUCA (Last Universal Common Ancestor) but, in either case, at least one viroid, or viroidlike RNA, must have been present in ancestors of animal cells to be joined to the protein-coding RNA. It follows that animal cells were, at that time, hospitable to viroids; and it is possible that other viroids existed in animal cells as well.

If so, why have they disappeared? Conceivably, the metabolism of animals, but not that of plants, changed with time, such as to make animals less hospitable for the survival of viroids, and they eventually became extinct—the only exception being HDV RNA, which, as an undoubtedly rare event, evolved to “find refuge” in the capsids of a virus (HBV) and thus became protected from the presumed damaging animal metabolism.

While apparently no evidence exists of viroid-inhibiting metabolites present only in particular hosts, not in others, such compounds have not been actively searched for and could easily have been overlooked—in which case, absence of a particular viroid in some, but not other hosts or host families, would not necessarily permit one to draw conclusions regarding the evolutionary age of that viroid.

## Conclusions

Both reviewers’ reports penetratingly and convincingly state reasons why my hypothesis faces severe difficulties and may be wrong. Why, if viroids are of primordial provenance, have none been isolated from cyanobacteria or from any other organisms in the evolutionary line older than angiosperms? Have they been sufficiently searched for in such “out-of-the-way” plants? Maybe not, because most viroid-investigating plant pathologists are, and have been, concerned with viroids’ agricultural, not evolutionary significance.

With these considerations in mind, and encouraged by both reviewers’ statements that my 1989 hypothesis is still alive, I propose that it should not be abandoned, but held in abeyance, awaiting future pertinent experimental evidence for or against it.

Sophisticated, newly conceived alternative hypotheses (see reviewers’ reports) convincingly claim that viroids could have evolved de novo at more recent than primordial times.

However, such hypotheses leave unexplained why extant viroids so “strikingly” display several crucial properties of posited primordial RNAs. Being common features of viroids, presence of these properties in extant viroids is unlikely to be a multiple coincidental occurrence.

Clearly, a decision between these opposite theories is not possible at this time; only considerable, dedicated new research could eventually solve the dilemma.

Hopefully, this paper will stimulate novel research, in which case both reviewers’ reports, with their original ideas, would be an essential help, by suggesting exciting new investigations, be there in line with Dr. Koonin’s novel protocol for studying a possible de novo origin of viroids, which, based on the early, seminal work of Dr. Sol Spiegelman, offers a viable alternative explanation for the origin of viroids, or Dr. Dolja’s similarly promising suggestions to the same effect. Such investigations may also help illuminate an early period in the evolution of life, a state of abiogenesis that, located between the first stage---the abiotic, chemical synthesis of amino acids and nucleotides, which is relatively well known [24]---and that of the third stage, the synthesis of primordial RNAs in the RNA world, which, at least in principle, is also understood, whereas the intermediate stage is almost totally unknown.

### Ethics approval and consent to participate

Not applicable.

### Consent for publication

Not applicable.

### Availability of data and materials

Not applicable.

## View reviewer comments for manuscript

### Valerian Dolja (Reviewer 1)

Reviewer Recommendation Term: Endorse publication

This manuscript is useful in attracting attention to poorly investigated problem of the viroids’ origin, but provides insufficient support to the concept of direct descent of viroids from primordial RNA world. I fully support Dr. Diener’s effort to revitalize discussion on the origins of viroids, the smallest known replicons that encode no proteins and possess ribozyme activity, thus recapitulating two features of the postulated primordial replicons of the RNA world. It should be emphasized, however, that extant viroids, as well as Hepatitis delta agent, lack an even more critical feature, i.e. ability to self-replicate, instead relying on polymerase proteins provided by the host. My other major reservations with the hypothesis of viroid’s direct descent from RNA world are as follows. First, to the best of our knowledge, viroid-like parasitic agents are found only in two types of organisms, higher plants (angiosperms) and vertebrates, but in none of their presumed ancestors, algae and more primitive animals, respectively. Furthermore, there is no data on any such agents in a vast variety of unicellular eukaryotes whose evolutionary history predates that of plants or animals by hundreds of millions of years. Worst yet, no viroid-like agents were reported in any of the prokaryotes, either archaea or bacteria that existed long before emergence of eukaryotes. Thus, according to author’s hypothesis, primordial viroid ancestors should have treaded a very narrow evolutionary path leading straight to land plants, but being eliminated from other organisms, even those that believed to be direct plant ancestors, such as Characean algae. To my opinion, a hypothesis allowing de novo origin of viroids in the higher plants, perhaps, via recombination between a pre-existing, cellular, ribozyme-possessing RNA with one that endowed the product with DNA-dependent RNA polymerase recognition signal, appears to be a simpler and thus more plausible scenario. This not to say that the original idea of Dr. Diener that viroids, as the smallest known autonomous replicons are most reminiscent of the RNA world replicons is not valid; it is, and very much so. I am not so fond of the hypothesis 2 for one simple reason: the data, supporting hypothesis 2 are not yet peer-reviewed, published, or independently confirmed. It feels premature to use these data until that happens. In conclusion, I am in favor of publishing this manuscript because the problem of viroids’ origin is extremely important and grossly under-investigated. The author’s hypotheses broaden the discussion and, hopefully, could stimulate the relevant experimental research.

Response to Dr. Dolja’s review of my manuscript

I wish to thank Dr. Dolja for his thoughtful and constructively critical review, as well as for his excellent suggestions for improving the manuscript. His idea of creating an alternative hypothesis to the primordial RNA origin of viroid-hypothesis, by allowing de novo origin of viroids in the higher plants via recombination of a preexisting, cellular, ribozyme-possessing RNA, with one that endowed the product with DNA-dependent RNA polymerase recognition signals—promises to be a made-to-order solution to the viroids’ serious descent dilemma. There would be left, however. a plausible explanation, why the de novo-created viroids “strikingly” resemble their posited primordial RNA-like features, which would now be bereft of functional significance.

Dr. Dolja is quite correct: the preliminary data, as well as hypothesis 2 in toto are not suitable for publication. I have deleted both, including mention of the unpublished data.

Theodor O. Diener.

## View reviewer comments for manuscript

### Eugene Koonin (Reviewer 2)

Reviewer Recommendation Term: Endorse publication

This Hypothesis article by Theodor Diener, the discoverer of viroids, revives his 25 year old hypothesis that viroids are living fossils of the primordial RNA World. The hypothesis is worth attention for two important reasons: first, it comes from the discoverer of these minimal replicons and will be of interest to many for that fact alone, and second, the idea of the primordial origin of viroids is a “natural” and as such, should be explicated, discussed and revisited. Indeed, among all known replicons, viroids come the closest to what one would envisage as a vestige of the RNA world: they are small RNA molecules that encode no proteins yet replicate autonomously and vigorously. Moreover, just as expected of putative survivors of the RNA World, viroids possess ribozyme activity that is required for their replication. These are striking arguments pro the hypothesis of the primordial origin of viroids. In fact, so obvious and (seemingly) persuasive are these arguments that, contrary to the rather surprising statement of Diener that his hypothesis had been ignored for 20 years, it had been lauded as a fundamentally important and best available scenario for the origin of viroids (e.g. Flores R, Gago-Zachert S, Serra P, Sanjuán R, Elena SF. Viroids: survivors from the RNA world? Annu Rev Microbiol. 2014; 68:395-414, and references therein). There are two serious contra arguments: first, viroids employ their ribozyme activity only for the processing of the replicative intermediates whereas replication itself is catalyzed by an elaborate host enzyme, the DNA-dependent RNA polymerase (nuclear or chloroplast), and second, the host range of viroids, to the best of our current knowledge, is limited to plants. The first counter-argument is not as damning as it might seem. Indeed, it would be unreasonable to expect that any RNA World survivor could retain the ancestral, RNA-only mode of replication which undoubtedly was much less efficient than any of the varieties of the modern, protein-catalyzed replication. The second counter-argument is harder to refute with as the limited host range of viroids implies numerous losses of viroids not only in animals (the focus of Diener’s discussion) but also in numerous lineages of bacteria, archaea and unicellular eukaryotes. This is a decidedly non-parsimonious evolutionary scenario (tens if not hundreds of evolutionary events as opposed to only one, the origin of viroids within ancestral plants) which is the main reason I hold the opinion that Diener's hypothesis is false and an alternative scenario for the origin of viroids should be sought (see below). First, however, I have to address Diener’s evidence that is deemed to be supportive of the primordial hypothesis. The first one comes from the mathematical model of Ma and colleagues (Diener’s Ref. 17) that is purported to demonstrate the plausibility of the hypothesis on the primordial origin of viroids. With all my respect for and interest and involvement in mathematical modeling of evolution, this does not come even close to testing the hypothesis. The model includes a variety of far-fetched assumptions (protein-less transcription, RNA cells and more) and at best is of interest as a conceptual exploration of replication processes in and RNA World that contained viroid-like molecules. It says nothing on the origin of the actual modern plant pathogens. Actually, I strongly doubt that any mathematical model, even a more realistic and carefully constructed one, could do anything substantial in that direction. The second line of evidence presented by Diener has to do with the purported replication of HDV in plant cells and of viroids in animal cells, in the presence of delta-antigen. Regrettably, this argument is based solely on unpublished experiments of Pelchat that are quoted in Ref. 22. This is troubling for purely formal reasons but more important, because in the absence of essential technical data, in particular, on the specificity of the stimulation of viroid replication in animal cells by delta-antigen, the reader cannot assess the validity of the claims. Assuming that these experiments are fully controlled and completely reproducible (a big assumption), such findings will provide us with a valuable insight into viroid-host interaction. However, even under this best case scenario, this will not buttress the primordial hypothesis at all. Indeed, it is completely reasonable to propose, as Diener does, that for some reasons, animal cells are inhospitable to viroids (or double-stranded or highly structured RNA molecules in general) and become permissive only in the presence of proteins with certain properties such as those of delta-antigen. Should this be the case, further molecular exploration of this system will be of considerable interest. However, this has no bearing on the origin of viroids. Furthermore, Diener assumes that, if there is one viroid-like agent (HDV) in animals, there should be more, and furthermore, that such agents should be ancestral to animals. This is, however, a non sequitur: why not horizontal transfer of a viroid from plants to mammals, e.g. via an insect vector? Actually, given the rarity of viroid-like agents in animals, I find this to be the most likely scenario. So what is the origin of viroids? We have no way of knowing but I think alternative scenarios that are more credible than the primordial one are not too difficult to conjure. In my brief discussion of such possibilities, I would like to first quote Diener: “Nevertheless, viroids seem to recapitulate the principle features of the selfish elements from the ancient RNA world”— thus leading to an internal contradiction, in that by one criterion (molecular properties), viroids “strikingly” appear to be descendants of primordial RNAs, whereas by another criterion (apparent evolutionary age), they clearly are not. To decide between them, I have conceived a new hypothesis.’ (the text in double quotes is from our paper with Valerian Dolja (Ref. 20). In this argument, there is a strong hidden assumption: ‘resembling’ = ‘descendants of’. Is this assumption valid? Definitely, viroids resemble primordial RNA genomes in some of the basic properties. Does this imply that the former actually descend from the latter? This depends on the complexity of the entity involved. In the case of viroids, the answer, I believe, is: not at all. As Diener correctly points out, citing the seminal work of Szathmary and Maynard Smith (Ref. 19), parasites inevitably evolve in any replicator system. Moreover, a universal trend in the evolution of parasites, with examples abounding, from viruses to parasitic worms, is genome reduction. There is one that might be especially relevant to the origin of viroids and hence worth citing, namely the classic early experiments of Spiegelman and colleagues on the in vitro evolution of RNA bacteriophages. In these serial transfer experiments, where phage encoded proteins did not contribute to genome replication given that the replicase was provided externally, the size of the genome rapidly dropped about 10-fold. What remained, was a minimal replicon, a RNA molecule of approximately 400 nucleotides, resembling viroids in size and the extent of secondary structure (Spiegelman S. An approach to the experimental analysis of precellular evolution. Q Rev Biophys. 1971 Aug;4(2):213-253; Mills DR, Kramer FR, Spiegelman S. Complete nucleotide sequence of a replicating RNA molecule. Science. 1973 Jun 1;180(4089):916-927). In other words, under the appropriate selective pressure, the RNA virus genome readily evolved into a small non-coding RNA that effectively retained only the signals for the replicase recognition. These experiments might serve as a model of the evolutionary processes that led to the emergence of viroids. The key intermediate could be satellite RNAs of plant viruses of so-called group 3 which are viroid-like circular RNA molecules that replicate via a rolling circle mechanism similar to that employed by viroids but utilizing the RNA-dependent RNA polymerase of the host virus (Rao AL, Kalantidis K. Virus-associated small satellite RNAs and viroids display similarities in their replication strategies. Virology. 2015 May;479-480:627-36, and references therein). Such satellite RNAs could have evolved from the viral RNA by genome reduction, losing the protein-coding genes, in a path mimicking Spiegelman’s experiments. Clearly, under this scenario, the signals for replicase recognition were already present in the ancestral RNA, so the evolution of the viroid-like satellite would only require the acquisition of a hammerhead ribozyme required for the circular RNA replication. Alternatively, both viroids and satellite RNAs could have evolved from small host RNA, for example, intronic ones, which would not involve the loss of protein-coding genes but would require acquisition of both the replicase recognition signals and the ribozyme. Importantly, it has been shown that the simple hammerhead ribozyme can easily evolve on multiple occasions (Salehi-Ashtiani K, Szostak JW. In vitro evolution suggests multiple origins for the hammerhead ribozyme. Nature. 2001 Nov 1; 414(6859):82-84). Thus, the path to the de novo evolution of viroids seems to be rather straightforward. Does the above argument falsify the primordial hypothesis? Certainly, not. To do so, it would be necessary to discover evolutionary intermediates of the de novo route of evolution. This seems to be a possibility but so far, there is no evidence of such intermediate forms. Moreover, it is easy to imagine new findings that would favor the primordial hypothesis, in particular, discovery of viroid-like agents in Cyanobacteria. However, in the absence of such evidence, I find the hypothesis of in planta origin of viroids to be more plausible that the primordial scenario. My position with regard to the hypotheses presented in the paper is discussed above. The author might want to cite some references where the hypothesis on the primordial origin of viroids is discussed and actually positively assessed. The claim in the current version of the manuscript, that this hypothesis had been ignored for decades, is inaccurate. This is particularly strange because the author fails to acknowledge the credit given by others to his own ideas. The use of unpublished data of other researchers is problematic (even though a permission is mentioned). I wonder if there is by now any publication, preprint or at least abstract that could be cited. In the very least, a formal acknowledgement is necessary. The reference list was apparently prepared manually and contains some errors and typos: Ref. 4 - page range cannot be correct Ref. 6 - year missing Ref. 8 - typo Ref. 10 - Mimivirus not Minivirus On several occasions, ‘hospital’ is used where it should be ‘hospitable’.

Response to Dr. Koonin’s review of my manuscript

I wish to express my deep gratitude to Dr. Koonin for honoring me with this very detailed, scientifically most informative and, last but not least, beautifully organized and written review, which stands by itself as a well documented essay in its own right. Needless to state, I shall not try to describe Dr. Koonin’s brilliant hypothesis of de novo origin of viroids in my revised manuscript, but only to refer the reader to its description in his review of my original manuscript—hopefully expecting, its timely publication.

But now to Dr. Koonin’s suggestions for improving my manuscript. Most importantly, I have deleted hypothesis 2 and all references to Pelchat’s preliminary results. Also, I assume a neutral attitude between the old primordial RNA and the new de novo origin theory, now that Dr.Koonin has convinced me that the former is probably false.

I am sorry, if I have failed to cite important, relevant publications. which may have been due to my relying on PubMed to keep me up-to-date, but have now learned that this source is not infallible. Thus, for example, PubMed failed to cite the one early effort to obtain more convincing arguments for the primordial RNA hypothesis, written by Cheles-Flores in1994 (Refs. 12 and 13). I have checked all 132 papers in Google Scholar, which cited my 1989 hypothesis paper. I determined that 34 were from the Ricardo Flores’ group, today arguably the foremost viroid-investigating group in existence (but whose members were not actively pursuing work relating to the origin of viroids, until their “review” of my 1989 hypothesis, which Dr. Koolin quotes in his review), 16 concerned Hepatitis Delta Virus, 76 were assorted reviews and papers not otherwise involved with viroid origin, and only 6 touched directly on viroid origin—5 of which I had already cited, leaving only the Cheles-Flores papers.

Theodor O. Diener

## Abbreviations

HBV: Hepatitis B virus
HDV: Hepatitis D (δ) virus
mRNA: messenger RNA
rRNA: ribosomal RNA
tRNA: transfer RNA
ncRNA: noncoding RNA
lncRNA: long noncoding RNA
